# Spinal gunshot wounds: A systematic review of the literature

**DOI:** 10.1016/j.xnsj.2025.100755

**Published:** 2025-06-21

**Authors:** Madhav Sankhyan, Sajni Amin, Kathleen S. Botterbush, Jorge F. Urquiaga, Emma Dwyer, Howard M. Place, Philippe J. Mercier, Najib El Tecle, Tobias A. Mattei

**Affiliations:** a Division of Neurological Surgery, Saint Louis University School of Medicine, 1008 S. Spring Avenue, 3rd Floor, St. Louis, MO 63110, United States; b Department of Neurosurgery, The University of Mississippi Medical Center, 2500 N. State St. Jackson, MS 39216-4505, United States; c Department of Orthopaedic Surgery & Rehabilitation Medicine, The University of Chicago, Duchossois Center for Advanced Medicine, MC 3079. 5758 S. Maryland Avenue, Dept. 4B, Chicago, IL 60637, United States; d Department of Orthopaedic Surgery, Saint Louis University School of Medicine, 1008 S. Spring Avenue, 1st Floor, St. Louis, MO 63110, United States; e Department of Neurological Surgery, Northwestern Medicine, 259 E. Erie Street, Chicago, IL 60611, United States

**Keywords:** Antibiotic prophylaxis, Complications, Gunshot wound, Management, Penetrating spinal cord injury, Surgery

## Abstract

**Background:**

The appropriate management of spinal gunshot wounds (GSWs) remains controversial. This systematic review presents an overview of the available scientific evidence in the literature regarding efficacy and complication rates of surgical management and antibiotic prophylaxis for spinal GSWs.

**Methods:**

A systematic review of PubMed/Medline was conducted, with a screening of articles for low-velocity civilian spinal GSWs. Collected information included patient demographics, injury description, management decision, outcomes of surgical versus conservative management, and the incidence of intra and extraspinal infections with prophylactic antibiotic use. Improvement was defined as the recovery of 1 or more ASIA levels. Statistical analysis involved chi-squared analysis and meta-analysis where feasible.

**Results:**

Thirty-four retrospective cohort studies were included. About 11% of patients were treated surgically whereas 89% were treated conservatively. Of the surgically managed patients, 88% received decompression, and only 11% received stabilization. Surgically managed patients had similar rates of neurological improvement in comparison to conservative treatment (95% CI –0.99, 13.45; p = .06), but 7% higher complication rate which was statistically significant (95% CI 0.66, 13.10; p = .02). Intraspinal infection rates were similar between the prophylaxis versus no prophylaxis groups (95% CI 1.12, 3.23; p = .35). However, the prophylaxis cohort had a higher rate of extraspinal infections (95% CI 34.82, 50.14; p < .01).

**Conclusions:**

An understanding of the factors which impact decision-making in spinal GSWs is of paramount importance. We found no statistically significant difference in neurological recovery between surgically and conservatively treated patients, although surgical patients had a statistically higher rate of complications. The possible deleterious effects of antibiotic prophylaxis on extraspinal infections warrants further research regarding confounding factors and which antibiotics regimens may be implicated.

## Introduction

Gunshots account for a significant portion of spinal cord injuries, partly due to the increasing use of low-velocity firearms (those with muzzle velocity of 1,000–2,000 ft/sec) in violent civilian crimes [[1], [2], [3], [4]]. According to the National Spinal Cord Injury Statistical Center, acts of violence—of which gunshots are the majority—cause approximately 14% of spinal cord injuries, ranking 3rd, behind only motor vehicle collisions and falls from height [[3], [4], [5]]. The incidence of penetrating spinal injuries (PSI) from gunshots also poses a significant economic burden to healthcare resources, with conservative estimated lifetime costs of $1.2 to 5.1 million per patient [5]. Despite their prevalence and socioeconomic burden, the optimal management of spinal gunshot wounds (GSW) remains controversial due to a paucity of published guidelines and the low quality of the scientific literature on the topic. Beyond initial resuscitation, treatment options may include surgical intervention versus conservative management and antibiotic prophylaxis. This systematic review explores the current utilization of these techniques and their outcomes to better inform future management and prognostication.

Initial management of PSIs from gunshots follows standard Advanced Trauma Life Support protocols (ATLS) [3,4,6], focusing on airway management and hemodynamic stability. According to the American College of Surgeons Trauma Quality Programs (ACS TQP) Best Practices Guidelines on spine injury, spinal immobilization is generally not required for most spinal gunshot wounds but may be continued for pain control or instability [7]. Importantly, clinicians should also recognize that overaggressive cardiopulmonary resuscitation maneuvers may contribute to worsening neurological function, especially in patients with unstable cervical and thoracolumbar spinal injuries. Once medically stabilized, spine surgeons evaluate the extent of the gunshot injury, considering several factors such as bullet size, trajectory, injury site, entry and exit wounds, associated visceral and vascular injuries (eg, vertebral artery injuries), vertebral column stability, and neurological deficits [3,4]. These factors are then considered when weighing the risks and benefits of surgical intervention.

Surgery is occasionally considered in cases of neurological deterioration, persistent cerebrospinal fluid (CSF) fistulae, retained bullet fragments at risk of migration, associated epidural hematoma with mass effect upon the neural elements, and mechanical instability, amongst others [8,9]. However, most spinal GSWs are not associated with gross spinal instability and thus do not require surgical stabilization [10,11]. In fact, some authors suggest that reported long-term instability in these patients may often be iatrogenic and mostly linked to poorly indicated decompressive laminectomies [3,11,12]. However, given the lack of definitive guidelines, higher-quality data are urgently needed to precisely determine the role of surgical intervention in spinal GSWs.

Similarly, the literature demonstrates conflicting recommendations regarding the need for antibiotic prophylaxis in civilian spinal GSWs. Some authors suggest immediate broad-spectrum coverage for 48 hours regardless of injury site and wound cultures; others recommend up to 14 days of antibiotic therapy [3,6,10,13,14]. This lack of consensus is partly due to the heterogeneity of spinal GSW patients. For example, patients with associated bowel perforation and other visceral injuries have a higher risk of infections and septic complications and may require longer antibiotic treatment regimens, unlike those with isolated spinal GSWs without visceral structure involvement [1,13,14].

Considering these inconsistent recommendations for surgical intervention and antibiotic prophylaxis in patients with spinal GSWs, the objectives of this systematic review are to (1) investigate the indications, efficacy, and complications of surgical management of patients with spinal GSWs and (2) explore the possible benefits, or lack thereof, of prophylactic antibiotic strategies in patients with spinal GSWs.

## Methods

A systematic review of the U.S. National Library of Medicine database was conducted through PubMed following PRISMA (Preferred Reporting Items for Systematic Reviews and Meta-Analyses) guidelines [15]. The Boolean terms “(spine OR spinal) AND gunshot” were used to identify relevant studies between 1966 (the earliest date of publications available in PubMed) and March 2021. Abstracts were manually reviewed to identify all articles reporting low-velocity civilian spinal GSWs, ultimately excluding military or combat-related spinal GSWs. Collected variables included patient demographics, clinical presentation, injury severity, management approach (surgical vs. conservative), complications rates, incidence and prophylactic treatment of spinal and nonspinal infections, and incidence and treatment of CSF leaks.

Initial screening criteria focused on retrospective cohort studies or randomized controlled trials, including abstracts of all articles and the full texts of open access articles. Exclusion criteria included all other articles, including—but not limited to—reviews, case reports, case series, letters, commentaries, editorials, bibliographies, and historical articles (Appendix A). We additionally excluded non-English articles, animal studies, subscription-based articles inaccessible through our institution and library system as a whole, articles reporting military and combat-related injuries, and articles reporting on high-velocity weapon injuries (>2,000 ft/sec). Articles, including the abstracts of subscription-based articles, were further screened for relevance.

Two independent researchers (SA and MS) individually reviewed each article. Articles were predominantly screened by information provided in the abstract, with full texts assessed if relevance was unclear based on the abstract alone. Articles which did not appear to be relevant to the objectives of this systematic review were subsequently excluded. When institutionally inaccessible articles appeared relevant, appropriate attempts were made to request access to the article for inclusion in our systematic review.

From the screened articles, the following information, when present, was collected: total number of cases; gender distribution; age; level of injury (cervical, thoracic, or lumbosacral); instituted management and comparative improvement between complete versus incomplete spinal cord injuries; total complications of surgery (decompression, instrumentation, wound debridement or foreign body retrieval) versus conservative management; incidence of spinal infections, extraspinal infections, pressure ulcers, neurogenic bladder, deep vein thromboses (DVTs)/pulmonary embolisms (PEs), neuropathic pain, and CSF leaks/fistulae. CSF leaks were defined as a compromise of the meningeal layer with CSF leakage to an unspecified area, while CSF fistulae were defined as CSF drainage to another internal body cavity. To reduce the impact of complications from other causes upon our analysis, complications occurring more than 1 month after the initial gunshot wound were excluded. Finally, patients who received steroids during their care were excluded from the analysis of infectious data.

For the purposes of this review, penetrating spinal injuries (PSIs) refer to any damage to the spinal column, regardless of whether the spinal canal was directly penetrated. Spinal infections were defined as meningitis, discitis, vertebral osteomyelitis, epidural abscesses, and paravertebral abscesses. Extraspinal infections were defined as pneumonia/empyema, urinary tract infections, acute pyelonephritis, acute cholecystitis, bacteremia/septicemia with positive blood cultures, peritonitis, urosepsis, colitis, abscesses (subcutaneous, abdominal, psoas), wound/surgical site infections or unspecified nonsurgical infections.

Our main statistical analyses were performed using chi-squared analysis, with a significance threshold of p = .05. 95% confidence intervals, with a corresponding Z score of 1.96, were calculated for the difference in proportions.

A meta-analysis with forest plots could not be performed for all studies because many of them did not provide direct comparisons of surgical versus conservative management, or antibiotic prophylaxis versus no prophylaxis, which would have been necessary for proper odds ratio calculations. Thus, the bulk of our data was represented as the difference in proportions. A few studies provided sufficient data enabling meaningful comparisons of improvement and complication rates between surgically and conservatively managed patients, allowing for a meta-analysis. For these eligible studies, odds ratios with 95% confidence intervals were calculated for dichotomous outcomes and pooled using a random-effects model. Statistical heterogeneity was evaluated using the I^2^ test, with I^2^ values over 50% suggesting substantial heterogeneity. Outcomes in the meta-analyses were pooled using weights from the inverse-variance method. Statistical significance was defined as a p < .05.

## Results

### ASIA grade improvement

Thirty-four articles were ultimately included in our systematic review, from an initial 936 search results (Appendices A and E) [9,[11], [12], [13],[15], [16], [17], [18], [19], [20], [21], [22], [23], [24], [25], [26], [27], [28], [29], [30], [31], [32], [33], [34], [35], [36], [37], [38], [39], [40], [41], [42], [43], [44]]. A total of 10,880 patients were included, with a mean age range of 15.6 to 56.2 years. Males comprised 87% of the total patient population. Of 2,320 injuries reported by spinal level, cervical and lumbosacral made up 26% each, whereas thoracic made up 48%. There were 2,391 patients who were categorized using the American Spinal Injury Association (ASIA) Impairment Scale (AIS) (Appendix B), ranging from ASIA A to ASIA E. For this study, we grouped patients into either complete spinal cord injury (namely ASIA A), which made up 56% of patients, and incomplete spinal cord injury (ASIA B to ASIA E), which made up 44% of patients.

Of 10,643 patients with reported treatment paradigms, 11% were treated surgically and 89% were treated conservatively. Of note—halo stabilization, where mentioned, was classified as conservative treatment. In the surgical cohort, 88% underwent decompression, 11% spinal stabilization, 3% wound debridement, and 10% foreign body or bullet removal.

Neurological recovery following surgical or conservative treatment was based on documented improvement in at least 1 ASIA level. Patients presenting as ASIA A had a significantly lower rate of improvement (at 6%), compared to 17% for ASIA B to ASIA D (p < .01, 95% CI –14.95% to –6.81%). As ASIA E patients are, by definition, neurologically intact, no improvement rate could not be quantified for them.

### Surgery

**Fig. 1:**
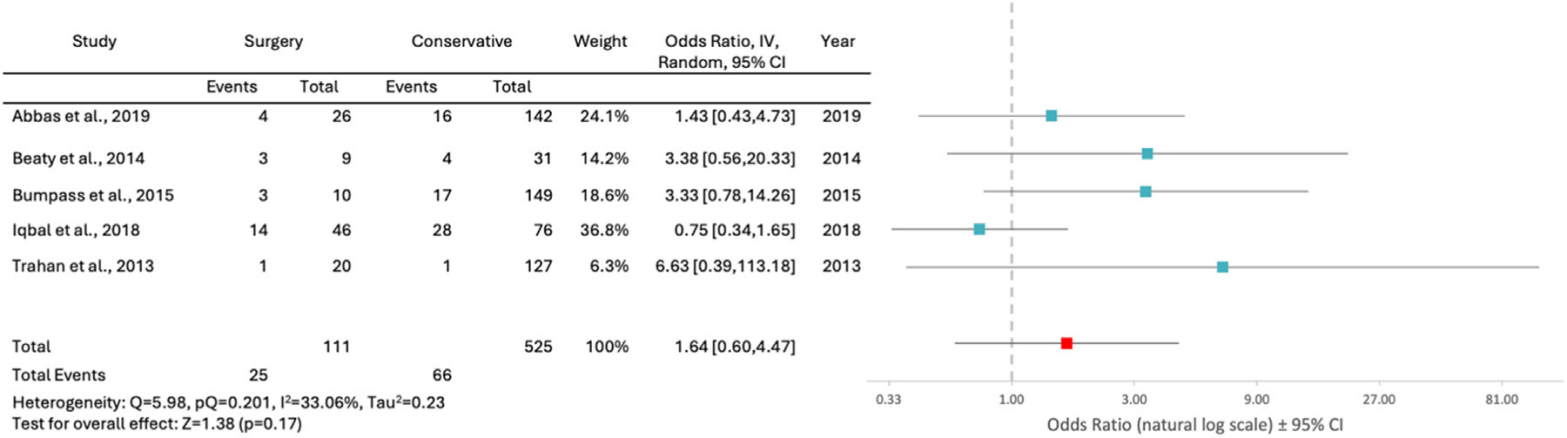
Forest plot comparing improvement after surgical and conservative management in patients with spinal gunshot wounds; CI, confidence interval; IV, inverse variance.

Of the patients in the surgical cohort, 19% improved 1 or more ASIA levels compared to 13% in the conservative management cohort. However, this 6% greater improvement in surgically managed patients compared to conservatively managed patients, was found to be non-statistically significant (p = .06, 95% CI −0.99% to 13.45%). Two of 21 patients who had spinal stabilization, and 4 of 43 patients who had decompression, showed neurological improvement. A random-effects model meta-analysis using 5 eligible studies also failed to show a statistically significant difference in the rate of improvement between surgical and conservative management (OR 1.64; 95% CI 0.60, 4.47; p = .17, I^2^ = 33.06%) (Fig. 1).

**Fig. 2:**
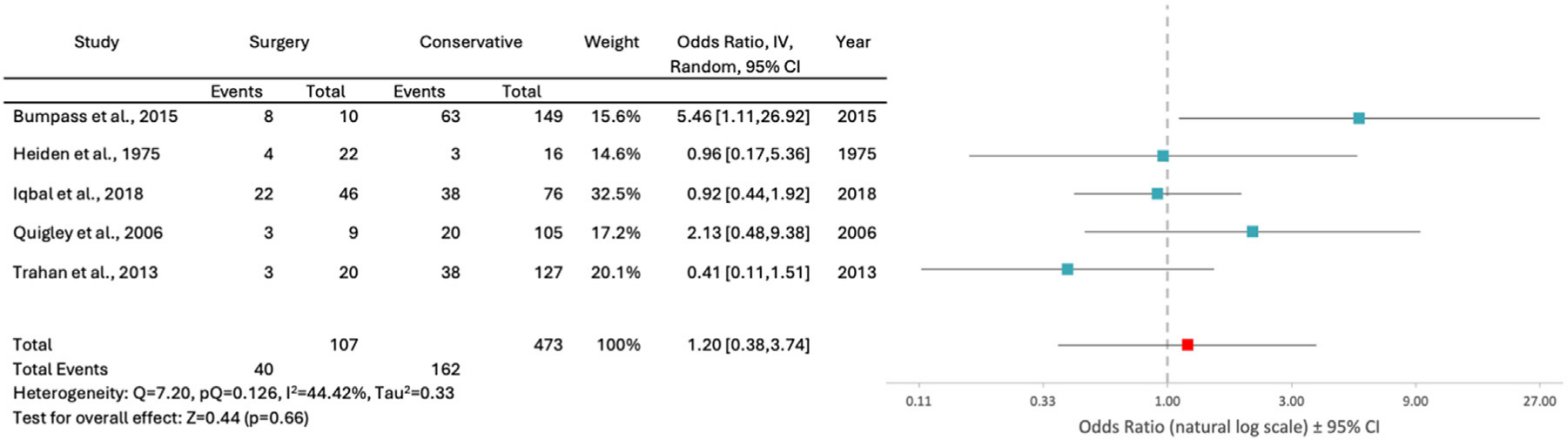
Forest plot comparing complication rates after surgical and conservative management in patients with spinal gunshot wounds; CI, confidence interval; IV, inverse variance.

Regarding complications of treatment, surgical patients had a 25% complication rate compared to 18% in conservatively managed patients. This 7% difference in complication rates between the 2 cohorts was found to be statistically significant (p = .02, 95% CI 0.66%–13.10%). However, a random-effects model meta-analysis using 5 studies eligible for meta-analysis found no significant difference in the complication rates between surgical and conservative management (OR 1.20; 95% CI 0.38, 3.74; p = .66, I^2^ = 44.42%) (Fig. 2). The most common complications were extraspinal infections (*n* = 550), with the 3 most common being UTIs (*n* = 231), pneumonias (*n* = 156) and wound/surgical site infections (*n* = 45). There was a total of 45 spinal infections reported. Other common complications included pressure ulcers (*n* = 150), DVT/PE (*n* = 69) and neurogenic bladder (*n* = 62).

### Antibiotic prophylaxis

Reported antibiotic prophylaxis durations ranged from 1 to 14 days with significant variability within and across studies. Some studies specified cephalosporins as the antibiotic of choice, while many simply stated broad spectrum coverage, and numerous others did not report choice of antibiotic altogether.

From a cohort of 903 patients, 735 received a prophylactic antibiotic regimen on presentation to the hospital. This cohort had a 2.2% spinal infection rate and 47% extraspinal infection rate. 40 patients received no antibiotics on presentation. They had no cases of spinal infections, and a 5% extraspinal infection rate. There were 1,082 patients for whom the prophylactic antibiotic use was not mentioned, and thus unknown. This cohort had a 2.8% spinal infection rate and 13.4% extraspinal infection rate. Finally, there were 306 patients for whom a prophylactic antibiotic treatment regimen was mentioned, but without report of how many actually received the antibiotics. This cohort had a 1% spinal infection rate and a 22.9% extraspinal infection rate.

Of the 2,163 patients in total, including those given antibiotics, those held, and those for whom status was unknown, there was a 2.3% rate of spinal infections and 26.2% rate of extraspinal infections. The difference in rates of spinal infections amongst the groups was not statistically significant for prophylaxis versus no prophylaxis (p = .35), prophylaxis versus unknown treatment regimen (p = .43), and prophylaxis versus unspecified prophylaxis (p = .19). However, the higher rate of extraspinal infections in the prophylaxis cohort was statistically significant (p < .01) for all 3 comparisons.

### CSF leaks

Twenty-nine (1.8%) CSF leaks and fistulae were reported out of a cohort of 1,592 patients. Ten leaks or fistulae were treated with open repair, and seventeen were treated with bedside repair (e.g. lumbar drains or bedside skin suturing). Five patients received open dural repairs, but still had persistent leakage that was treated with bedside closure. The treatment for the remaining cases was not reported. There were no unresolved cases of CSF leaks. Additionally, in the surgical cohort, fifteen durotomies without subsequent development of CSF leaks or fistulae were reported. One intraoperative durotomy was reported as iatrogenic.

## Discussion

### Pathophysiology of spinal GSWs

Spinal GSWs are complex injuries, influenced by various factors that affect management. This study focused on civilian GSWs (muzzle velocity 1,000–2,000 ft/second), although high velocity rifle injuries (2,000–3,000 ft/second) typical of military combat, also cause significant neurological morbidity [4]. Other ballistic factors influencing tissue damage and treatment choice include bullet size, composition, projectile path, and firearm-to-target distance (Appendix D) [4].

Bullet composition, specifically, can alter management. Although rare, the American College of Surgeons Trauma Quality Programs Best Practices Guidelines recommend monitoring lead levels every 3 months for the first year in patients with retained lead bullets. Chelation or bullet removal should be considered if levels rise or remain above 5µg/dL [7,45]. Experimental studies also suggest that intradural copper fragments may be more neurotoxic than lead or aluminum while extradural fragments were not associated with significant risks [46,47]. Although this rationale may support a decision to remove intradural copper bullet fragments, surgical risks and uncertainty of fragment composition complicate the actual decision-making in the clinical setting.

With regard to bullet location, routine removal of intracanal fragments is not always advised. However, one study demonstrated significant improvement in motor recovery with intracanal bullet removal from levels T12 to L4 [48]. Studies also suggest that progressive neurological deficits from retained intracanal bullets at any level may warrant surgical removal [46].

Biological factors are a second category of variables used to describe GSWs. These are the main variables we focused on during this review and include: level of spine injury, presence of retained fragments causing compressive or toxic effects, tissue contamination, and development of spinal instability, among others [4]. Our systematic review suggests that most spinal GSW patients are males. Nearly half of the injuries occur at the thoracic level, and nearly half are complete injuries. A systematic review by Sidhu et al. [49] reported similar findings: 87% male population, and 49% presented with thoracic injuries, 30% with cervical and 21% lumbosacral. However, they also reported a wide range (13%–78%) of complete spinal cord lesions, with nearly 70% of their cervical GSWs resulting in complete lesions.

Our data did not differentiate ASIA A (complete) versus ASIA B-ASIA E (incomplete) injuries by level of spinal injury. However, we did find a significant 11% increased rate of improvement in patients with incomplete spinal cord injuries. Although, by itself the clinical relevance of an 11% improvement is unknown, this knowledge, alongside the overall low recovery rates, may help guide expectations and prognostication. Notably, prior studies have demonstrated worse motor and sensory prognosis with complete SCIs compared to incomplete injuries. Bumpass et al. reported that 31% of patients with incomplete SCIs improved at least 1 ASIA level by their last follow-up (the mean follow-up duration was 13.6 months) [8].

Rehabilitation is also known to play a key role in patients with GSWs. The Functional Independence Measure (FIM) is a tool that can determine a patient’s rehabilitation needs- patients with incomplete SCIs may benefit from intensive rehabilitation aimed at regaining function, whereas complete SCIs often require assistive devices and other compensatory strategies to achieve independence as functional recovery is less likely [30].

### Conservative versus surgical management of spinal GSWs

Based on our review, most spinal GSW patients (89%) are treated conservatively. Among those who underwent surgery, decompression was the most common procedure (88%), while spinal stabilization was performed in only 11%. Surgical patients showed a 19% rate of neurological improvement, 6% higher than conservative treatment, but this difference was not statistically significant (p = .06, 95% CI −0.99% to 13.45%). This difference was also not statistically significant in our meta-analysis of 5 studies (OR 1.64; 95% CI 0.60, 4.47; p = .17, I^2^ = 33.06%) (Fig. 1). Even if there were a statistically signficant difference in favor of surgical treatment, there is still the possibility that such an apparent benefit could be a reflection of a selection bias, as patients with incomplete deficits and surgically amenable findings (eg, epidural hematoma or bone fragments in spinal canal) are more likely to undergo surgery. Thus, it is reasonable to conclude that, although possible, surgical intervention is probably only indicated in a very small subset of patients with spinal GSWs.

It should also be highlighted that in very rare circumstances, surgery may be the best strategy to reverse a course of acute neurological deterioration in patients without a significant neurological deficit at presentation [50]. This is supported by the American College of Surgeons Trauma Quality Programs Best Practice Guidelines, which suggest that penetrating injuries to the cervical and thoracic spine do not typically show neurologic improvement after surgery; thus, surgical intervention should be reserved for cases of ongoing compression, neurologic deterioration, CSF leaks and overall instability [7].

Spine surgeons assess biomechanical spinal instability using factors such as the presence of pedicle fractures, bilateral facet involvement, posterior ligamentous complex integrity, spinal misalingment and degree of vertebral body compromise. These factors are accounted for in major classification systems like the Subaxial Cervical Spine Injury Classification and Severity Scale (SLICS), and the Thoracolumbar Injury Classification and Severity Score (TLICS). Though useful to assess spinal instability and guide surgical decision-making in blunt spinal trauma, these tools are not validated for PSIs, and no alternatives exist [51]. Thus, while these bony and ligamentous injury patterns may suggest the presence of spinal instability, spine surgeons should bear in mind that these are predictive factors which have not been specifically studied in the context of PSIs.

In general, it is very difficult to draw definitive conclusions about the role of surgical treatment in patients with spinal GSWs from the available data in the literature. This is further complicated by the observed 7% increase in complication rates (from 25% in surgical patients compared to 18% in conservatively managed patients, p = .02, 95% CI 0.66%–13.10%). While this difference was found to be statistically significant, its clinical relevance when considering a surgery with proper indications is somewhat difficult to contextualize. To better evaluate the accuracy of these results, a meta-analysis was conducted; however it did not find the difference in the rate of complications to be statistically significant (OR 1.20; 95% CI 0.38, 3.74; p = .66, I^2^ = 44.42%) (Fig. 2). This result may reflect the limited number of studies in the meta-analysis and their moderate heterogeneity as demonstrated by an I^2^ value of 44.42%.

In the end, knowledge of the most common complications in patients with spinal GSWs as revealed by our study (UTIs, pneumonias and wound/surgical site infections) is still highly relevant as it may help clinicians anticipate patients’ broader care needs. However, due to limited data in the studies included, it remains unclear whether these complications stem from the primary injury, or surgery (or lack thereof), so future studies with more granular data delineating the etiology of observed complications are necessary.

Ultimately, numerous variables must be considered on an individual basis prior to determining whether surgical or conservative management is appropriate for patients. While a randomized controlled trial would clarify management, the heterogeneity of spinal GSW patients makes this approach largely impractical. A trial in a smaller subgroup of patients (for example in those who are neurologically intact but present with an intradural bullet segment) is conceivable, but would likely face major sample size limitations even across multiple centers.

### Antibiotic prophylaxis in spinal GSWs

The use of antibiotics in the management of patients with spinal GSWs remains controversial, with no consensus on treatment regimens nor efficacy and risks of prophylactic treatment. Some studies recommend ≥7 days of broad-spectrum prophylaxis immediately following injury for all patients [6,52], whereas others found no increase in spinal or neurological infection rates with shorter (<48 hour) regimens [3,52]. Trans-intestinal or other transluminal injuries complicate matters further, with prophylactic treatment being more strongly indicated in such a scenario, and recommendations ranging from 2 to 14 days including anaerobic coverage [3,6]. The lack of high-level evidence to guide treatment prompted our investigation of the literature on efficacy rates and complications of prophylactic antibiotic treatment in patients with spinal GSWs.

In our study, the prophylaxis group and the no prophylaxis group had a 2.2% and 0% rate of spinal infections, respectively, a non-significant difference (p = .35), suggesting that antibiotic prophylaxis has no significant effect on the incidence of spinal infections. However, extraspinal infections were significantly higher in the antibiotic prophylaxis group (47% vs. 5%; p < .01). This significant difference may reflect factors such as the breeding of resistance in pathogens causing hospital-acquired infections, disruption of native flora, or differences in hospitalization durations which contribute to nosocomial infections. Unfortunately, a meta-analysis for these data was unattainable due to a lack of studies that investigated the outcomes of antibiotic prophylaxis in our population of interest. Based on available data, it is likely that routine antibiotic prophylaxis may have a deleterious effect in terms of extraspinal infections and should only be considered when absolutely indicated, such as in the case of intestinal or other intra-luminal injuries

Of note, because of the poor quality of available data we were unable to perform a subgroup analysis involving transluminal and non-transluminal GSWs. Ultimately, a randomized controlled trial with treatment and control groups stratified by transluminal involvement, is needed to provide a definitive recommendation on the indication of antibiotic prophylaxis.

Notably, in 2024, the American Association for the Surgery of Trauma (AAST) Critical Care Committee took a significant step in the direction of providing clear practice guidelines by releasing a clinical consensus document on the use of antibiotic prophylaxis following a variety of traumatic injuries [53]. For PSIs, they recommend a short course of prophylactic antibiotics lasting ≤48 hours, using first and second-generation cephalosporins, ampicillin-sulbactam, piperacillin-tazobactam, or clindamycin with a second-generation cephalosporin [54]. The only special consideration listed for penetrating spine injury was gastrointestinal involvement, specifically trans-colonic disruptions. In cases with trans-intestinal injuries, which increases the risk for spinal and paraspinal infections, the literature remains highly variable. The general consensus, primarily based on clinical judgement and older studies, has been to extend the course of antibiotics to last up to 14 days [[53], [54]]. However, recent studies have favored shorter courses, with statistically similar rates of infection between the standard 48-hour course and longer courses, possibly suggesting that longer courses in patients with trans-intestinal injuries may be unnecessary [[35], [36],[55], [56]]. Ultimately, no formal guideline was issued for PSIs with intestinal involvement; the committee instead recommended “an adequately powered, prospective study that evaluates the necessity, type, and length of therapy” [53].

### Dural tears and CSF fistulae in spinal GSWs

Dural tears and subsequent formation of CSF fistulae are possible complications of spinal surgery. Kalidindi et al. [57] estimate dural tears occur in 1% to 17% of spine surgeries. They also estimated the incidence of late-presenting dural tears, defined as late presenting CSF leaks without intraoperatively recognized dural tears, to be 0.06 to 0.83 percent [57].

Finally, as observed in this series, most patients with spinal GSWs for whom surgery is indicated, will have strong risk factors for a dural tear secondary to the initial GSW itself, such as presence of a sharp bony fragment in the canal or a bullet trajectory tangential to the spinal canal. We found an overall incidence of CSF leaks and fistulae of 1.8 percent, although there was considerable variability in how each study defined leaks and fistulae.

Generally, early recognition of CSF leaks is crucial due to the possibility of serious side effects, including wound infection, meningitis, intracranial hemorrhage or neurological deficits [58,59]. Recommended treatments for CSF leaks range from lumbar drainage and bed rest to direct dural repair [57,58,60], with some authors also suggesting prophylactic antibiotic use [57]. In our study, we were interested in exploring the need for antibiotic prophylaxis and infection rates of control and treatment groups. Unfortunately, the data available was limited and often did not report on infection rates. Ultimately, further research exploring this association is required to determine the need for antibiotic prophylaxis in confirmed cases of CSF leaks secondary to spinal GSWs.

### Limitations and future directions

This study had several limitations. First, every article we included was a retrospective cohort study with no control group. There was also considerable heterogeneity among the studies, with variable inclusion and exclusion criteria, neurological assessments and outcome measures.

Subgroup analysis by spinal segment and level (ie, injury to the spinal cord versus cauda equina), was also not possible due to insufficient reporting. Unfortunately, inconsistent reporting of outcomes in the included studies also made it impossible to comparatively evaluate long-term neurological outcomes in complete versus incomplete spinal cord injuries across both the surgical and conservative treatment groups. Future cohort studies should consider evaluating this in more detail. Moreover, although complications of treatment cohorts were comprehensively reported in some studies, they were only briefly mentioned in others.

Regarding antibiotic treatment, some studies provided exact treatment regimens for individual patients, whereas others failed to mention the duration, route of administration, type of coverage, or dosage employed. Therefore, our analysis of the data was grouped into broad categories of patient cohorts. These may have had large confounding effects on the data for which we have not accounted.

Additionally, our search was restricted to the PubMed database. Although providing broad coverage of the scientific literature relevant to our topic, particularly when applying our targeted search strategy with Boolean terms, this single-database approach may limit the generalizability of our findings. Future studies could attempt to utilize databases beyond PubMed, like EMBASE, Scopus and Cochrane.

Given these various limitations of included studies, assigning individual levels of evidence and completing individual assessments of risk of bias assessments for each study was deemed to add little value. In fact, this could be even more misleading as it is likely that all retrospective cohort studies would have been classified as having the same level of evidence, despite the fact that they were not found to be equal in terms of the quality of the evidence they provided. Ultimately, the final data summarized in this systematic review are classified as Level III evidence and translates to a Class III strength of recommendation [61].

## Conclusions

Several ballistic and biological factors play an important role in determining the best therapeutic approach for patients with spinal GSWs and ultimately renders this group of patients quite heterogeneous in terms of the clinical evolution, neurological outcome and risks of complications.

Our data suggest that most patients are managed conservatively. Surgical intervention may be indicated in a smaller subgroup of patients (such as patients with spinal instability or those with incomplete deficits with mass effect) but was associated with a significantly higher rate of complications. Thus, the decision to pursue surgical intervention should be tailored and individualized to each patient while carefully considering the possible benefits and risks of surgical intervention.

Similarly, according to our data, the use of prophylactic antibiotics showed no improvement in the rate of spinal infections, but a statistically significant increase in extraspinal infections. This strongly suggests that prophylactic antibiotic therapy should not be part of the standard protocol for management of patients with spinal GSWs. This may differ in cases of trans-luminal injuries, cases of known ongoing infection, or in the context of CSF leaks and fistulae.

Ultimately, the low quality of the available data in the literature suggests a pressing need for further high-quality studies on this topic as well as clear guidelines from professional societies.

## Declaration of competing interest

The authors declare that they have no known competing financial interests or personal relationships that could have appeared to influence the work reported in this paper.

## Appendix Supplementary materials

Image, application 1

Image, application 2

Image, application 3

Image, application 4

Image, application 5
