# Supplementary material for: Spinal gunshot wounds: A systematic review of the literature
Source: N Am Spine Soc J. 2025 Jun 21;23:100755. doi: 10.1016/j.xnsj.2025.100755 (PMC12318342; doi:10.1016/j.xnsj.2025.100755)

Appendix A

PRISMA [Preferred Reporting Items for Systematic Reviews and Meta-Analyses] Flow Diagram of systematic review of studies on gunshot wounds to the spine. Screened, excluded and included articles are outlined. No automation tools were used in the exclusion process, and all records were excluded by hand.^15^


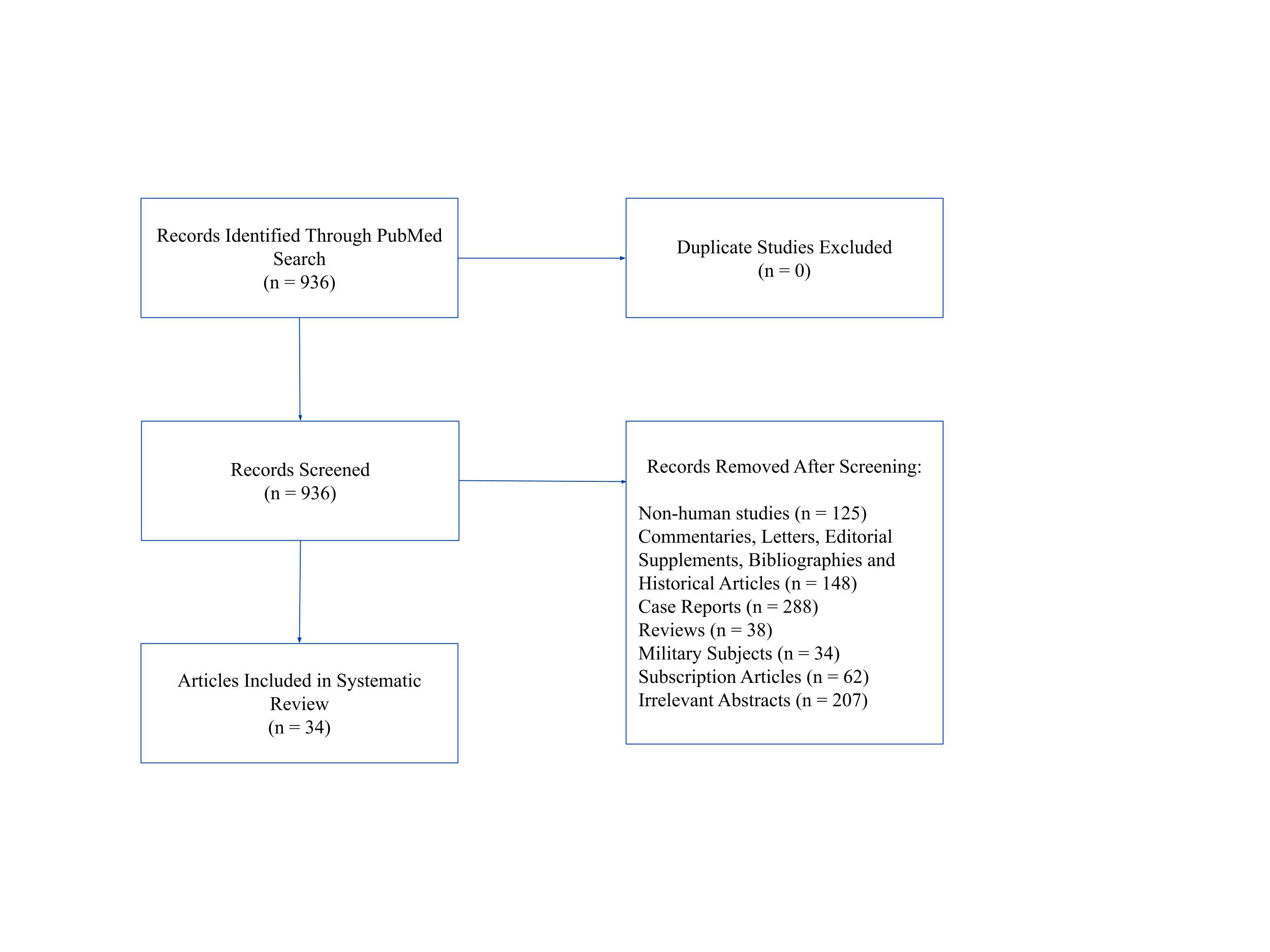

Supplement: Supplementary file 1 [file mmc1.docx]
