# Supplementary material for: Spinal gunshot wounds: A systematic review of the literature
Source: N Am Spine Soc J. 2025 Jun 21;23:100755. doi: 10.1016/j.xnsj.2025.100755 (PMC12318342; doi:10.1016/j.xnsj.2025.100755)
Supplement: Supplementary file 2 [file mmc2.docx]

Appendix B

ASIA Impairment Scale (AIS)

| **ASIA Impairment Scale** | | |
| --- | --- | --- |
| **A** | Complete | No motor, no sensory, no sacral sparing |
| **B** | Incomplete | No motor, sensory only |
| **C** | Incomplete | 50% of muscles LESS than grade 3 |
| **D** | Incomplete | 50% of muscles MORE than grade 3 |
| **E** | Normal | Motor and sensory function are normal |
