# Supplementary material for: Spinal gunshot wounds: A systematic review of the literature
Source: N Am Spine Soc J. 2025 Jun 21;23:100755. doi: 10.1016/j.xnsj.2025.100755 (PMC12318342; doi:10.1016/j.xnsj.2025.100755)
Supplement: Supplementary file 3 [file mmc3.docx]

Appendix C

Summary Data of All Articles Combined

Table C.1. Patient/Injury Demographics

| Total Cases | 10880 |
| --- | --- |
| Males | 7931 |
| Females | 1224 |
| Unspecified Sex | 1725 |
| Cervical | 662 |
| Thoracic | 1117 |
| Lumbosacral | 600 |
| Complete | 1338 |
| Incomplete | 1053 |
| Incomplete Cervical/Thoracic/Lumbosacral | 136/217/161 |

Table C.2. Treatment Cohorts

| Conservatively Managed | 9436 |
| --- | --- |
| Surgically Managed | 1207 |
| Decompression | 960/1091 |
| Stabilization | 121/1104 |
| Debridement/Infection Control | 37/1104 |
| Foreign Body Removal | 110/1104 |

(x/y = number of cases/total number of patients in cohort reporting variable x)

Table C.3. Patient Outcomes

| Complete SCI Improved | 24/412 |
| --- | --- |
| Incomplete SCI Improved | 78/467 |
| Conservative Improved | 66/525 |
| Surgery Improved | 25/133 |
| Decompression Improved | 4/35 |
| Stabilization Improved | 2/21 |

*Abbreviations:* SCI, spinal cord injury

Table C.4. Complications

| Surgery Complications | 55/221 |
| --- | --- |
| Conservative Complications | 163/905 |
| Spinal Infections | 45 |
| Extraspinal Infections | 550 |
| Pneumonia/Empyema | 156/550 |
| UTI | 231/550 |
| Wound/Surgical Site Infections | 45/550 |
| Pressure Ulcers | 150 |
| Neurogenic Bladder | 62 |
| Neuropathic Pain | 45 |
| DVT/PE | 69 |
| GI Complications | 32 |
| CSF Fistulae | 29/1592 |
| Durotomies | 15 |

*Abbreviations:* CSF, cerebrospinal fluid; DVT, deep vein thrombosis; GI, gastrointestinal; PE, pulmonary embolism; UTI, urinary tract infection

Table C.5. Antibiotic Prophylaxis Outcomes

| Treatment Cohort | Patients | Spinal Infections | Extraspinal Infections |
| --- | --- | --- | --- |
| Abx Administered | 735/903 | 16 | 349 |
| Abx Administered | Unspecified/306 | 3 | 70 |
| Abx Not Administered | 40 | 0 | 2 |
| Abx Treatment Not Mentioned | 1082 | 30 | 145 |

*Abbreviations:* abx, antibiotics
