# Supplementary material for: Spinal gunshot wounds: A systematic review of the literature
Source: N Am Spine Soc J. 2025 Jun 21;23:100755. doi: 10.1016/j.xnsj.2025.100755 (PMC12318342; doi:10.1016/j.xnsj.2025.100755)
Supplement: Supplementary file 4 [file mmc4.docx]

Appendix D

Ballistic and biological factors affecting the management and prognosis of spinal gunshot wounds. Table adapted from Jaiswal et al., Asian Spine Journal 2013.

| **Ballistic Factors** |
| --- |
| Type of firearm |
| Size of the projectile/bullet |
| Composition of the projectile/bullet (jacketed vs unjacketed) |
| Speed of projectile/bullet |
| Distance between firearm and target |
| **Biological Factors** |
| Site of spine injury |
| Vertebral column instability |
| CSF leak/dural tear |
| Retained metallic/bony fragment in canal |
| Contaminated tissue in canal |
| Associated hollow viscus, lung or vascular injuries |

*Abbreviations:* CSF, cerebrospinal fluid
