# Supplementary material for: Spinal gunshot wounds: A systematic review of the literature
Source: N Am Spine Soc J. 2025 Jun 21;23:100755. doi: 10.1016/j.xnsj.2025.100755 (PMC12318342; doi:10.1016/j.xnsj.2025.100755)
Supplement: Supplementary file 5 [file mmc5.docx]

Appendix E

Summary Data of Individual Articles Included in Systematic Review

Table E.1. Study-type and Participant Demographics

| Authors & Year | Title | Type of Study | Study Location | Number of Patients Included in the Original Study | Number of Cases Included in Systematic Review | Males Included in Systematic Review | Females Included in Systematic Review |
| --- | --- | --- | --- | --- | --- | --- | --- |
| Abbas et al., 2018 | Gunshot Acquired Spinal Cord Injury in Civilians | Retrospective | Turkey | 168 | 168 | 154 (91.7%) | 14 (8.3%) |
| Aryan et al., 2005 | Gunshot wounds to the spine in adolescents | Retrospective | USA | 60 | 60 |  |  |
| Beaty et al., 2014 | Cervical spine injury from gunshot wounds | Retrospective | USA | 40 | 40 | 37 (92.5%) | 3 (7.5%) |
| Bumpass et al., 2015 | An update on civilian spinal gunshot wounds: treatment, neurological recovery, and complications | Retrospective | USA | 159 | 159 | 147 (92.5%) | 12 (7.5%) |
| Cornwell et al., 2001 | Thoracolumbar immobilization for trauma patients with torso gunshot wounds: is it necessary? | Retrospective | USA | 1000 | 141 |  |  |
| DuBose et al., 2009 | The role of routine spinal imaging and immobilisation in asymptomatic patients after gunshot wounds | Retrospective | USA | 327 | 327 |  |  |
| Eftekhary et al., 2016 | Overutilization of bracing in the management of penetrating spinal cord injury from gunshot wounds | Retrospective | USA | 396 | 396 | 376 (94.9%) | 20 (5.1%) |
| Gutierrez et al., 2020 | Penetrating Spinal Column Injuries (pSI): An Institutional Experience with 100 Consecutive Cases in an Urban Trauma Center | Retrospective | USA | 100 | 95 |  |  |
| Guzelkucuk et al., 2016 | Spinal cord injury resulting from gunshot wounds: a comparative study with non-gunshot causes | Retrospective | Turkey | 1043 | 57 | 47 (82.5%) | 10 (17.5%) |
| Heary et al., 1997 | Steroids and gunshot wounds to the spine | Retrospective | USA | 254 | 254 | 231 (90.9%) | 23 (9.1%) |
| Heiden et al., 1975 | Penetrating gunshot wounds of the cervical spine in civilians. Review of 38 cases | Retrospective | USA | 38 | 38 |  |  |
| Inaba et al., 2011 | Clinical examination is highly sensitive for detecting clinically significant spinal injuries after gunshot wounds | Prospective | USA | 112 | 12 |  |  |
| Iqbal et al., 2018 | Gunshot Spinal Injury: Factors Determining Treatment and Outcome | Retrospective? | Pakistan | 150 | 122 | 117 (95.9%) | 5 (4.1%) |
| Klein et al., 2005 | Spine injuries are common among asymptomatic patients after gunshot wounds | Retrospective | USA | 228 | 228 |  |  |
| Kumar et al., 1998 | Low-velocity gunshot injuries of the spine with abdominal viscus trauma | Retrospective | USA | 13 | 13 | 12 (92.3%) | 1 (7.7%) |
| Le Roux et al., 2005 | Gunshot injuries of the spine--a review of 49 cases managed at the Groote Schuur Acute Spinal Cord Injury Unit | Retrospective | South Africa | 49 | 49 | 38 (77.6%) | 11 (22.4%) |
| Levy et al., 1996 | Use of methylprednisolone as an adjunct in the management of patients with penetrating spinal cord injury: outcome analysis | Retrospective | USA | 252 | 252 | 236 (93.7%) | 16 (6.3%) |
| Lustenberger et al., 2011 | Unstable cervical spine fracture after penetrating neck injury: a rare entity in an analysis of 1,069 patients | Retrospective | USA | 1069 | 56 |  |  |
| McCunniff et al., 2017 | Operative Versus Nonoperative Management of Civilian Gunshot Wounds to the Spinal Cord: Novel Use of the Functional Independence Measure for Validated Outcomes | Retrospective | USA | 104 | 104 | 95 (91.3%) | 9 (8.7%) |
| Medzon et al., 2005 | Stability of cervical spine fractures after gunshot wounds to the head and neck | Retrospective | USA | 81 | 19 |  |  |
| Mirovsky et al., 2005 | Complete paraplegia following gunshot injury without direct trauma to the cord | Retrospective | Israel | 26 | 26 |  |  |
| Nwosu et al., 2017 | Surgical Management of Civilian Gunshot-Induced Spinal Cord Injury: Is It Overutilized? | Retrospective | USA | 489 | 489 |  |  |
| Putzke et al., 2001 | Gunshot versus nongunshot spinal cord injury: acute care and rehabilitation outcomes | Retrospective | USA | 212 | 212 | 189 (89.2%) | 23 (10.8%) |
| Quigley et al., 2006 | The role of debridement and antibiotics in gunshot wounds to the spine | Retrospective | USA | 114 | 114 | 106 (93.0%) | 8 (7.0%) |
| Rabinowitz et al., 2012 | Infectious complications in GSW's through the gastrointestinal tract into the spine | Retrospective | USA | 51 | 51 | 48 (94.1%) | 3 (5.9%) |
| Rhee et al., 2006 | Cervical spine injury is highly dependent on the mechanism of injury following blunt and penetrating assault | Retrospective | USA |  | 168 |  |  |
| Sajid et al., 2020 | Gunshot injury to spine: An institutional experience of management and complications from a developing country | Retrospective | Pakistan | 40 | 40 | 37 (92.5%) | 3 (7.5%) |
| Schubl et al., 2016 | Cervical spine immobilization may be of value following firearm injury to the head and neck | Retrospective | USA | 156 | 36 | 34 (94.4%) | 2 (5.6%) |
| Secer et al., 2015 | Relationship of biological factors to survival in spinal gunshot injuries | Retrospective | Turkey | 110 | 110 | 105 (95.5%) | 5 (4.5%) |
| Smith et al., 2003 | Spinal cord injury caused by gunshot wounds: the cost of rehabilitation | Retrospective | USA | 47 | 47 | 44 (93.6%) | 3 (6.4%) |
| Soni et al., 2020 | Regional and Institutional Practice Variations in Decompressive Spine Surgery for Patients with Penetrating Spinal Injury in the United States | Retrospective | USA | 6632 | 6632 | 5599 (84.4%) | 1032 (15.6%) |
| Trahan et al., 2013 | Gunshot wounds to the spine in post-Katrina New Orleans | Retrospective | USA | 147 | 147 | 135 (91.8%) | 12 (8.2%) |
| Velmahos et al., 1994 | Gunshot wounds of the spine: should retained bullets be removed to prevent infection? | Retrospective | South Africa | 153 | 153 | 144 (94.1%) | 9 (5.9%) |
| Yashon et al., 1970 | Prognosis and management of spinal cord and cauda equina bullet injuries in sixty-five civilians | Retrospective | USA | 65 | 65 |  |  |

Table E.2. Injury Sustained

| Authors & Year | Cervical Spine | Thoracic Spine | Lumbosacral Spine | Complete SCI/ASIA - A | Incomplete SCI/ASIA B-E |
| --- | --- | --- | --- | --- | --- |
| Abbas et al., 2018 | 42 (25.0%) | 48 (28.6%) | 33 (19.6%) | 45 (26.8%) | 123 (73.2%) |
| Aryan et al., 2005 | 12 (20.0%) | 31 (51.7%) | 17 (28.3%) | 34 (56.7%) | 26 (43.3%) |
| Beaty et al., 2014 | 40 (100%) |  |  | 28 (70.0%) | 12 (30.0%) |
| Bumpass et al., 2015 | 45 (28.3%) | 54 (34.0%) | 60 (37.7%) | 40 (25.2%) | 108 (67.9%) |
| Cornwell et al., 2001 |  |  |  | 73 (51.8%) | 58 (41.1%) |
| DuBose et al., 2009 |  |  |  |  |  |
| Eftekhary et al., 2016 | 103 (26.0%) | 229 (57.8%) | 64 (16.2%) | 280 (70.7%) | 116 (29.3%) |
| Gutierrez et al., 2020 |  |  |  |  |  |
| Guzelkucuk et al., 2016 | 14 (24.6%) | 37 (64.9%) | 6 (10.5%) | 33 (57.9%) | 24 (42.1%) |
| Heary et al., 1997 | 76 (29.9%) | 116 (45.7%) | 62 (24.4%) | 190 (74.8%) | 64 (25.2%) |
| Heiden et al., 1975 |  |  |  | 25 (65.8%) | 9 (23.7%) |
| Inaba et al., 2011 | 5 (41.7%) | 6 (50.0%) | 4 (33.3%) |  |  |
| Iqbal et al., 2018 | 38 (31.1%) | 48 (39.3%) | 36 (29.5%) | 48 (39.3%) | 74 (60.7%) |
| Klein et al., 2005 |  |  |  |  |  |
| Kumar et al., 1998 |  |  |  |  |  |
| Le Roux et al., 2005 | 13 (26.5%) | 24 (49.0%) | 12 (24.5%) | 38 (77.6%) | 8 (16.3%) |
| Levy et al., 1996 | 51 (20.2%) | 158 (62.7%) | 43 (17.1%) | 108 (42.9%) | 144 (57.1%) |
| Lustenberger et al., 2011 |  |  |  |  |  |
| McCunniff et al., 2017 | 30 (28.8%) | 53 (51.0%) | 21 (20.2%) | 67 (64.4%) | 37 (35.6%) |
| Medzon et al., 2005 | 19 (100%) |  |  |  |  |
| Mirovsky et al., 2005 |  |  |  |  |  |
| Nwosu et al., 2017 |  |  |  |  |  |
| Putzke et al., 2001 |  |  |  | 123 (58.0%) | 89 (42.0%) |
| Quigley et al., 2006 | 51 (44.7%) | 42 (36.8%) | 85 (74.6%) | 43 (37.7%) | 27 (23.7%) |
| Rabinowitz et al., 2012 | 0 (0%) | 16 (31.4%) | 37 (72.5%) |  |  |
| Rhee et al., 2006 |  |  |  |  |  |
| Sajid et al., 2020 | 9 (22.5%) | 20 (40.0%) | 11 (27.5%) | 25 (62.5%) | 15 (37.5%) |
| Schubl et al., 2016 |  |  |  |  |  |
| Secer et al., 2015 | 42 (38.2%) | 50 (45.5%) | 18 (16.4%) | 77 (70.0%) | 33 (30.0%) |
| Smith et al., 2003 | 10 (21.3%) | 27 (57.4%) | 11 (23.4%) |  |  |
| Soni et al., 2020 |  |  |  |  |  |
| Trahan et al., 2013 | 40 (27.2%) | 53 (36.1%) | 54 (36.7%) | 61 (41.5%) | 86 (58.5%) |
| Velmahos et al., 1994 | 22 (14.4%) | 105 (68.6%) | 26 (17.0%) |  |  |
| Yashon et al., 1970 |  |  |  |  |  |

*Abbreviations:* ASIA, American Spinal Injury Association; SCI, spinal cord injury

Table E.3. Treatment Cohorts and Patient Outcomes

| Authors & Year | Conservatively Managed | Surgically Managed | Laminectomy/ Decompression | Fusion/  Stabilization | Debridement/  Infection Control/  Washouts | Bullet/ Foreign Body Retrieval | Improvement Asia A | Improvement Asia B-E | Outcome (Surgery)  (Improve ≥ 1 ASIA Level) | Outcome (Conservative)  (Improve ≥ 1 ASIA Level) |
| --- | --- | --- | --- | --- | --- | --- | --- | --- | --- | --- |
| Abbas et al., 2018 | 142 (84.5%) | 26 (15.5%) | 21 | 5 | 0 | 8 | 0 (0%) | 20 (16.3%) | 4 (15.4%) | 16 (11.3%) |
| Aryan et al., 2005 | 60 (100%) | 0 (0%) | 0 | 0 | 0 | 0 |  |  |  |  |
| Beaty et al., 2014 | 31 (77.5%) | 9 (22.5%) | 3 | 7 | 0 | 0 | 3 (10.7%) | 4 (33.3%) | 3 (33.3%) | 4 (12.9%) |
| Bumpass et al., 2015 | 149 (93.7%) | 10 (6.3%) | 5 | 7 | 2 | 3 | 6 (15.0%) | 14 (13.0%) | 3 (30.0%) | 17 (11.4%) |
| Cornwell et al., 2001 | 133 (94.3%) | 8 (5.7%) | 2 | 2 | 0 | 4 |  |  |  |  |
| DuBose et al., 2009 | 325 (99.4%) | 2 (0.6%) | 2 | 0 | 0 | 0 |  |  | 0 (0%) |  |
| Eftekhary et al., 2016 | 396 (100%) | 0 (0%) | 0 | 0 | 0 | 0 |  |  |  |  |
| Gutierrez et al., 2020 | 83 (87.4%) | 12 (12.6%) | 8 | 6 | 0 | 0 |  |  |  |  |
| Guzelkucuk et al., 2016 | 34 (59.6%) | 23 (40.4%) |  |  |  |  |  |  |  |  |
| Heary et al., 1997 | 217 (85.4%) | 37 (14.6%) | 32 | 5 | 0 | 0 | 0 (0%) | 11 (17.2%) |  |  |
| Heiden et al., 1975 | 16 (42.1%) | 22 (57.9%) | 22 | 0 | 16 | 0 |  |  |  |  |
| Inaba et al., 2011 | 11 (91.7%) | 1 (8.3%) | 0 | 0 | 0 | 1 |  |  | 0 (0%) |  |
| Iqbal et al., 2018 | 76 (62.3%) | 46 (37.7%) |  |  |  |  | 15 (31.3%) | 27 (36.5%) | 14 (30.4%) | 28 (36.8%) |
| Klein et al., 2005 | 213 (93.4%) | 15 (6.6%) | 14 | 0 | 0 | 12 |  |  |  |  |
| Kumar et al., 1998 |  |  |  |  |  |  |  |  |  |  |
| Le Roux et al., 2005 | 32 (65.3%) | 17 (34.7%) | 0 | 6 | 0 | 11 |  |  |  |  |
| Levy et al., 1996 | 236 (93.7%) | 16 (6.3%) | 16 | 6 | 0 | 0 |  |  |  |  |
| Lustenberger et al., 2011 | 54 (96.4%) | 2 (3.6%) | 0 | 2 | 0 | 0 |  |  | 0 (0%) |  |
| McCunniff et al., 2017 | 81 (77.9%) | 23 (22.1%) | 23 | 0 | 1 | 23 |  |  |  |  |
| Medzon et al., 2005 | 16 (84.2%) | 3 (15.8%) | 3 | 3 | 0 | 0 |  |  |  |  |
| Mirovsky et al., 2005 | 7 (26.9%) | 19 (73.1%) | 19 | 0 | 0 | 0 |  |  |  |  |
| Nwosu et al., 2017 | 402 (82.2%) | 87 (17.8%) | 42 | 33 | 0 | 33 |  |  |  |  |
| Putzke et al., 2001 | 171 (80.7%) | 34 (16.0%) |  |  |  |  |  |  |  |  |
| Quigley et al., 2006 | 105 (92.1%) | 9 (7.9%) | 8 | 1 | 9 | 0 |  |  |  |  |
| Rabinowitz et al., 2012 | 49 (96.1%) | 2 (3.9%) | 0 | 2 | 0 | 0 |  |  |  |  |
| Rhee et al., 2006 | 159 (94.6%) | 9 (5.4%) | 0 | 9 | 0 | 0 |  |  | 0 (0%) |  |
| Sajid et al., 2020 | 27 (67.5%) | 13 (32.5%) |  | 5 | 3 | 0 |  |  |  |  |
| Schubl et al., 2016 | 3 (8.3%) | 0 (0%) | 0 | 0 | 0 | 0 |  |  |  |  |
| Secer et al., 2015 | 66 (60.0%) | 13 (11.8%) | 0 | 13 | 5 | 1 |  |  |  |  |
| Smith et al., 2003 | 39 (83.0%) | 8 (17.0%) | 8 | 0 | 0 | 0 |  |  | 0 (0%) |  |
| Soni et al., 2020 | 5956 (89.8%) | 676 (10.2%) | 676 | 0 | 0 | 0 |  |  |  |  |
| Trahan et al., 2013 | 127 (86.4%) | 20 (13.6%) | 11 | 9 | 0 | 6 | 0 (0%) | 2 (2.3%) | 1 (5.0%) | 1 (0.8%) |
| Velmahos et al., 1994 |  |  |  |  | 1 | 8 |  |  |  |  |
| Yashon et al., 1970 | 20 (30.8%) | 45 (69.2%) | 45 | 0 | 0 | 0 |  |  |  |  |

*Percentage of each surgical procedure performed is not provided as some patients required more than one surgical procedure, thus calculating a percentage would be misleading

*Abbreviations:* ASIA, American Spinal Injury Association

Table E.4. Secondary Complications

| Authors & Year | Complications of Surgery | Complications of Conservative Management | Pressure Ulcer | Neurogenic Bladder | DVT/PE | Neuropathic Pain, Back Pain | CSF/Dural Leak/Fistula | Spinal Infection | Wound/  Local/  Surgical Site Infection | Pneumonia/  Empyema/  Effusion | Extraspinal Infection | UTI | GI Complications |
| --- | --- | --- | --- | --- | --- | --- | --- | --- | --- | --- | --- | --- | --- |
| Abbas et al., 2018 |  |  | 20 | 18 | 8 | 2 |  | 3 | 2 | 8 |  |  |  |
| Aryan et al., 2005 |  |  |  |  |  |  |  |  | 5 |  |  |  |  |
| Beaty et al., 2014 |  |  |  |  |  |  | 3 |  |  |  |  |  |  |
| Bumpass et al., 2015 | 8 (80%) | 63 (42.3%) | 18 |  | 17 | 21 | 1 | 2 |  | 16 | 19 | 20 |  |
| Cornwell et al., 2001 |  |  |  |  |  |  |  |  |  |  |  |  |  |
| DuBose et al., 2009 |  |  |  |  |  |  |  |  |  |  |  |  |  |
| Eftekhary et al., 2016 |  |  |  |  |  |  |  |  |  |  |  |  |  |
| Gutierrez et al., 2020 | 2 (16.7%) |  |  |  |  |  | 1 |  | 1 |  |  |  |  |
| Guzelkucuk et al., 2016 |  |  |  |  |  |  |  |  |  |  |  |  |  |
| Heary et al., 1997 |  |  |  |  |  |  |  | 5 |  |  | 31 |  | 15 |
| Heiden et al., 1975 | 4 (18.2%) | 3 (18.8%) |  |  | 1 |  | 1 | 2 | 1 |  |  |  |  |
| Inaba et al., 2011 |  |  |  |  |  |  |  |  |  |  |  |  |  |
| Iqbal et al., 2018 | 22 (47.8%) | 38 (50.0%) | 12 |  | 6 |  | 3 | 14 | 3 | 12 |  | 14 |  |
| Klein et al., 2005 |  |  |  |  |  |  |  |  |  |  |  |  |  |
| Kumar et al., 1998 |  |  |  |  |  |  |  |  |  |  |  |  |  |
| Le Roux et al., 2005 |  |  | 6 |  | 1 |  |  | 3 |  | 6 |  |  |  |
| Levy et al., 1996 |  |  |  |  | 14 |  |  | 1 | 0 | 81 |  | 144 |  |
| Lustenberger et al., 2011 |  |  |  |  |  |  |  |  |  |  |  |  |  |
| McCunniff et al., 2017 |  |  | 20 |  | 10 |  | 4 |  | 1 |  | 17 | 47 | 17 |
| Medzon et al., 2005 |  |  |  |  |  |  |  |  |  |  |  |  |  |
| Mirovsky et al., 2005 |  |  |  |  |  |  |  |  |  |  |  |  |  |
| Nwosu et al., 2017 | 8 (9.2%) | 0 (0%) |  |  |  |  | 4 |  |  |  |  |  |  |
| Putzke et al., 2001 |  |  | 5 |  |  |  |  |  |  |  |  |  |  |
| Quigley et al., 2006 | 3 (33.3%) | 20 (19.0%) |  |  |  |  |  | 5 | 23 |  |  |  |  |
| Rabinowitz et al., 2012 | 1 (50.0%) |  |  |  |  |  |  | 1 | 6 | 6 | 31 | 6 |  |
| Rhee et al., 2006 |  |  |  |  |  |  |  |  |  |  |  |  |  |
| Sajid et al., 2020 | 4 (30.8%) | 0 (0%) | 12 |  |  |  |  |  |  |  | 7 |  |  |
| Schubl et al., 2016 | 0 (0%) | 1 (33.3%) |  |  |  |  |  |  |  |  |  |  |  |
| Secer et al., 2015 |  |  |  |  |  |  | 12 | 6 |  |  | 6 |  |  |
| Smith et al., 2003 |  |  | 28 | 44 |  | 22 |  |  |  |  |  |  |  |
| Soni et al., 2020 |  |  |  |  |  |  |  |  |  |  |  |  |  |
| Trahan et al., 2013 | 3 (15.0%) | 38 (30.0%) | 6 |  | 3 |  |  |  | 2 | 15 |  |  |  |
| Velmahos et al., 1994 |  |  | 23 |  | 9 |  |  | 3 | 1 | 12 | 7 |  |  |
| Yashon et al., 1970 |  |  |  |  |  |  |  |  |  |  |  |  |  |

*Percentage of each individual complication is not provided as each patient may have had multiple complications, thus calculating a percentage would be misleading

*Abbreviations:* CSF, cerebrospinal fluid; DVT, deep vein thrombosis; GI, gastrointestinal; PE, pulmonary embolism; UTI, urinary tract infection
